# Production and expression of recombinant anti-V3 scFvs from HIV-1 clade C infected Indian patient

**DOI:** 10.1186/1742-4690-9-S1-P3

**Published:** 2012-05-25

**Authors:** Rajesh Kumar, Raiees Andrabi, Ashutosh Tiwari, Somi Sankaran Prakash, Naveet Wig, Durgashree Dutta, Anurag Sankhyan, Lubina Khan, Subrata Sinha, Kalpana Luthra

**Affiliations:** 1 All India Institute of Medical Sciences, New Delhi, India

## Introduction

Neutralizing antibodies are an important component of the humoral immune response directed against viral infections So far the few available anti HIV-1 broadly neutralizing antibodies have a limited breadth and potency against clade C viruses. More than 50% of the HIV-1 infections worldwide belong to clade C. Clade C is the most prevalent subtype in India.

## Materials and methods

A human scFv phage display library was constructed from the peripheral blood mononuclear cells of an HIV-1 Clade C infected Indian patient, having a good titre of serum neutralizing antibodies. Diversity of the scFv library was checked by sequencing and DNAfingerprinting analysis of randomly selected clones from the preselected library. One round of biopanning was done against V3 peptide of clade C and clade B. Single chain fragments were checked for their soluble expression in E. coli HB2151 and purfied using Ni+2 affinity colums. Specificity of the soluble scFvs was checked by indirect ELISA. Single chain fragments were checked for their stability in different agents like 30%DMSO. 4M NaCl, pH 2-11.

## Results

50 clones were randomly selected after biopanning and they were checked for their binding to V3C and V3B peptides. In phage ELISA 15 out of 50 clones showed binding to both the V3 peptides. A 32kDa band was observed in polyacrylamide gel electrophoresis as predicted. The expressed product was confirmed by Western blot analysis using anti His Tag antibody. Specificity of the purified scFvs was confirmed by their binding to V3 peptides and no reactivity against other unrelated peptides. Further these scFvs displayed a stable binding to V3 peptides in different denaturing agents.

## Conclusions

This is the first study to generate human anti-V3 scFvs against HIV-1 clade C. Further characterization of these scFvs for their neutralization potential will help identify unique and shared epitopes responsible for neutralization of clade C and non clade C viruses.

